# *Camellia sinensis* phytochemical profiling, drug-likeness, and antibacterial activity against gram-positive and gram-negative bacteria: *in vitro* and *in silico* insights

**DOI:** 10.3389/fchem.2025.1555574

**Published:** 2025-03-11

**Authors:** Farouk Boudou, Amal Belakredar, Ahcene Keziz, Huda Alsaeedi, David Cornu, Mikhael Bechelany, Ahmed Barhoum

**Affiliations:** ^1^ Department of Biology, Faculty of Sciences, Djillali Liabes University of Sidi-Bel-Abbes, Sidi Bel-Abbès, Algeria; ^2^ Department of Biotechnology, Faculty of Natural Sciences and Life, University of Mostaganem Abdelhamid Ibn Badis, Mostaganem, Algeria; ^3^ Department of Physics, Physics, and Chemistry of Materials Laboratory University of M’sila, M’Sila, Algeria; ^4^ Department of Chemistry, College of Science, King Saud University, Riyadh, Saudi Arabia; ^5^ Institut Européen des Membranes, IEM, UMR 5635, ENSCM, CNRS, University Montpellier, Montpellier, France; ^6^ Functional Materials Group, Gulf University for Science and Technology (GUST), Mubarak Al-Abdullah, Kuwait; ^7^ NanoStruc Research Group, Chemistry Department, Faculty of Science, Helwan University, Cairo, Egypt; ^8^ Chemical and BioPharmaceutical Sciences, Technological University Dublin, Dublin, Ireland

**Keywords:** antibacterial activity, Camellia sinensis, molecular docking, molecular dynamics, phenolic compounds, HOMO-LUMO energy gap

## Abstract

**Background:**

*Camellia sinensis* extracts have a rich phytochemical profile and therapeutic properties. The plant contains bioactive compounds, such as catechins, flavonoids, and phenolic acids, which are associated with various health benefits, including antioxidant, anti-inflammatory, and anticancer activities.

**Aim:**

To investigate the bioactive potential of a *Camellia sinensis* extract, particularly its antibacterial activity against Gram-positive and Gram-negative bacteria and its drug-like properties.

**Method:**

Phenolic compounds in *C. sinensis* extract were identified and quantified using high-performance liquid chromatography (*HPLC*). Its antibacterial activity was assessed against both Gram-positive (*Staphylococcus aureus*) and Gram-negative bacteria (*Pseudomonas aeruginosa* and *Escherichia coli*). Drug-likeness, toxicity, and molecular properties of the identified compounds were investigated using computational approaches. Additionally, binding affinities of selected compounds were predicted via molecular docking to elucidate potential antibacterial mechanisms.

**Results:**

*HPLC* identified caffeic acid (10.32 mg/g), epigallocatechin gallate (*EGCG*, 8.74 mg/g), syringic acid (6.21 mg/g), and quercetin (15.29 mg/g). Antibacterial activity testing revealed inhibition zones ranging from 10.62 mm for Gram-negative *E. coli* to 18.65 mm for Gram-positive *S. aureus*, comparable to gentamicin (19.42 mm). Molecular docking predicted that *EGCG* (−9.8 kcal/mol) was the most potent compound against Gram-negative *P. aeruginosa* RNase PH, followed by quercetin (−8.7 kcal/mol). Drug-likeness modeling indicated favorable profiles for most compounds, although *EGCG* violated Lipinski’s rule due to its molecular weight (458.4 g/mol). Density Functional Theory analysis revealed significant variations in electronic properties among the selected compounds, with quercetin exhibiting the smallest HOMO-LUMO gap (2.31 eV), suggesting high reactivity. MD simulations confirmed the stability of the *EGCG*-protein complex, with RMSD values (∼2.5–3.0 Å), reduced RMSF at key residues, and stable Rg (∼18–20 Å).

**Discussion:**

The results highlight that *C. sinensis* is a valuable source of bioactive phenolic compounds with promising antibacterial properties against both Gram-positive and Gram-negative bacteria, particularly *EGCG*. Quercetin, the most abundant compound, showed better chemical stability (higher HOMO-LUMO gap), but its lower binding affinity suggests that *EGCG* is a more effective therapeutic candidate. Moreover, the antibacterial activity of these compounds positions them as potential alternatives to conventional antibiotics. Future research should focus on *in vivo* validation, structure-activity optimization, and formulation development to improve bioavailability and clinical applicability.

## 1 Introduction

Bacterial infections are a global health issue that concerns millions of people each year. For instance, *Escherichia coli* can cause severe urinary tract infections and life-threatening conditions, such as hemolytic uremic syndrome that has been linked to contaminated food (e.g., the 2011 outbreak in Germany caused by enterohemorrhagic *E. coli)* (Muniesa et al., 2012). Similarly, *Staphylococcus aureus*, including methicillin-resistant *S. aureus* (MRSA), can cause surgical site infections and community-acquired pneumonia, particularly in healthcare settings (Weese and Prescott, 2021). *Pseudomonas aeruginosa* causes infections in burns and individuals with cystic fibrosis. It also shows resistance to critical antibiotics, leading to difficult-to-treat cases of ventilator-associated pneumonia (Tacconelli et al., 2018). These pathogens, characterized by their adaptability and resistance mechanisms, have rendered conventional antibiotics less effective, necessitating the search for alternative therapies.

One promising alternative is the use of natural compounds derived from medicinal plants. Among these, *Camellia sinensis* (used to produce tea) stands out for its abundant phenolic content and well-documented health benefits, including antimicrobial activity (Zhao et al., 2022). Phenolic compounds found in *C. sinensis,* such as epigallocatechin gallate (EGCG) and quercetin, display significant activity against antibiotic-resistant strains. For example, EGCG enhances the efficacy of β-lactam antibiotics against MRSA by disrupting the bacterial cell wall (Zhao et al., 2022). Quercetin inhibits bacterial biofilm formation, a critical factor in antibiotic resistance, particularly in *P. aeruginosa* infections (Kim et al., 2021). These findings highlight *C. sinensis* potential as a natural source of bioactive compounds against drug-resistant bacteria.

*In silico* studies play a crucial role in the modern drug discovery pipeline by providing detailed insights into the interaction mechanisms between bioactive compounds and bacterial targets. For example, molecular docking studies showed that EGCG binds strongly to penicillin-binding proteins, with binding affinities comparable to those of standard antibacterial drugs (Zhao et al., 2021). Similarly, computational analyses revealed that quercetin can inhibit quorum sensing, a bacterial communication system crucial for virulence and resistance, particularly in *P. aeruginosa* (Shariati et al., 2024). These *In silico* data support the antibacterial potential of phytochemicals and also provide a framework for optimizing their structures to enhance their activity.

This study evaluated the antibacterial potential of a *C. sinensis* extract against clinically significant pathogens (*E. coli*, *S. aureus*, and *P. aeruginosa*), representing both Gram-positive and Gram-negative bacteria, using *in vitro* antibacterial assays and *in silico* approaches. After high-performance liquid chromatography (*HPLC*) analysis to identify and quantify key phenolic compounds in the extract, antibacterial testing (agar diffusion assays) was used to measure the extract inhibition zones. Molecular docking simulations were employed to assess the binding affinities of bioactive compounds (*EGCG* and quercetin) to bacterial target proteins, shedding light on their mechanisms of action. Additionally, drug-likeness and toxicity evaluations were conducted to assess the compound potential for therapeutic development. The *C. sinensis* extract was chosen because of its known health benefits, particularly its antimicrobial, antioxidant, and anti-inflammatory properties. This study is crucial for exploring natural plant-based compounds as alternatives to conventional antibiotics for the management of multidrug-resistant Gram-positive and Gram-negative bacteria. It contributes to the development of safer, more effective treatments for bacterial infections. By integrating experimental and computational techniques, this study aims to provide a comprehensive understanding of *C. sinensis* antibacterial properties, paving the way for future clinical applications.

## 2 Experimental

### 2.1 Plant extraction

Dried, finely powdered *C. sinensis* leaves (10 g), labeled as EL BERRAD^®^ Special Chunmee Green Tea from China, obtained from a local market, in Saida City, Algeria. The extract was prepared by maceration in methanol (100 mL) for 48 h. The sample was continuously stirred, and methanol was replaced after 24 h to maximize compound extraction. Then, the macerate was filtered using Whatman No. 1 filter paper and concentrated to dryness using a rotary evaporator under reduced pressure at a temperature not exceeding 40°C to avoid degradation of heat-sensitive compounds. The resulting dry extract was stored in an airtight container at 4°C.

### 2.2 Phytochemical profiling

For phytochemical profiling, the dry extract was dissolved in methanol (1 mg/mL) and filtered through a 0.45 µm membrane filter to remove residual particulates. Then, the filtrate was analysed by HPLC using a UV/Visible multiwavelength detector. The separation was performed on a Hypersil ODS C18 reversed-phase column (250 mm × 4.6 mm, 5 µm particle size) at a flow rate of 1 mL/min. The mobile phase consisted of acetonitrile (solvent A) and water (solvent B) with 0.2% sulfuric acid (v/v), using a gradient elution program with the following conditions: 0–10 min, 10% A, 90% B; 10–18 min, 30% A, 70% B; 18–28 min, 50% A, 50% B. Phenolic compounds were identified by comparing their retention times and UV spectra with those of known standards (quercetin, EGCG, and caffeic acid).

### 2.3 *In vitro* antibacterial activity

*Camellia sinensis* leaf extract antibacterial activity was evaluated using the agar diffusion method against *E. coli* ATCC 25922 (Gram-negative), *S. aureus* ATCC 25923 (Gram-positive), and *P. aeruginosa* ATCC 27853 (Gram-negative). Bacterial strains were cultured on nutrient agar plates at 37°C for 24 h and then suspended in sterile saline solution to achieve a concentration of approximately 10^8^ CFU/mL, calibrated to the 0.5 McFarland turbidity standard. Sterile Mueller-Hinton agar (20 mL) was poured into Petri dishes, and the bacterial suspension was inoculated on the agar surface with a sterile swab to ensure even distribution. Wells (6.0 mm diameter) were created on the inoculated agar surface using a sterile cork borer. A volume of 50 µL of *C. sinensis* extract in methanol (5,000 μg/mL, 2,500 μg/mL, 1,250 μg/mL, or 625 μg/mL) was added to each well. Negative control wells were filled with 50 µL of methanol, and positive control wells with 50 µL of known antibacterial agents (50 µg gentamicin, 30 µg pipemidic acid, or 20 µg cefazolin), incubated at 37°C for 24 h, the zones of inhibition were measured (in millimetres) using a calliper. The antibacterial activity was expressed as the diameter of the clear zone around each well, and the minimum inhibitory concentration (MIC) was determined based on the lowest extract concentration that gave a clear zone of inhibition. All assays were performed in triplicate to ensure reproducibility of the results.

### 2.4 *In silico* antibacterial activity modelling

#### 2.4.1 Selection of potential ligands

**FIGURE 1:**
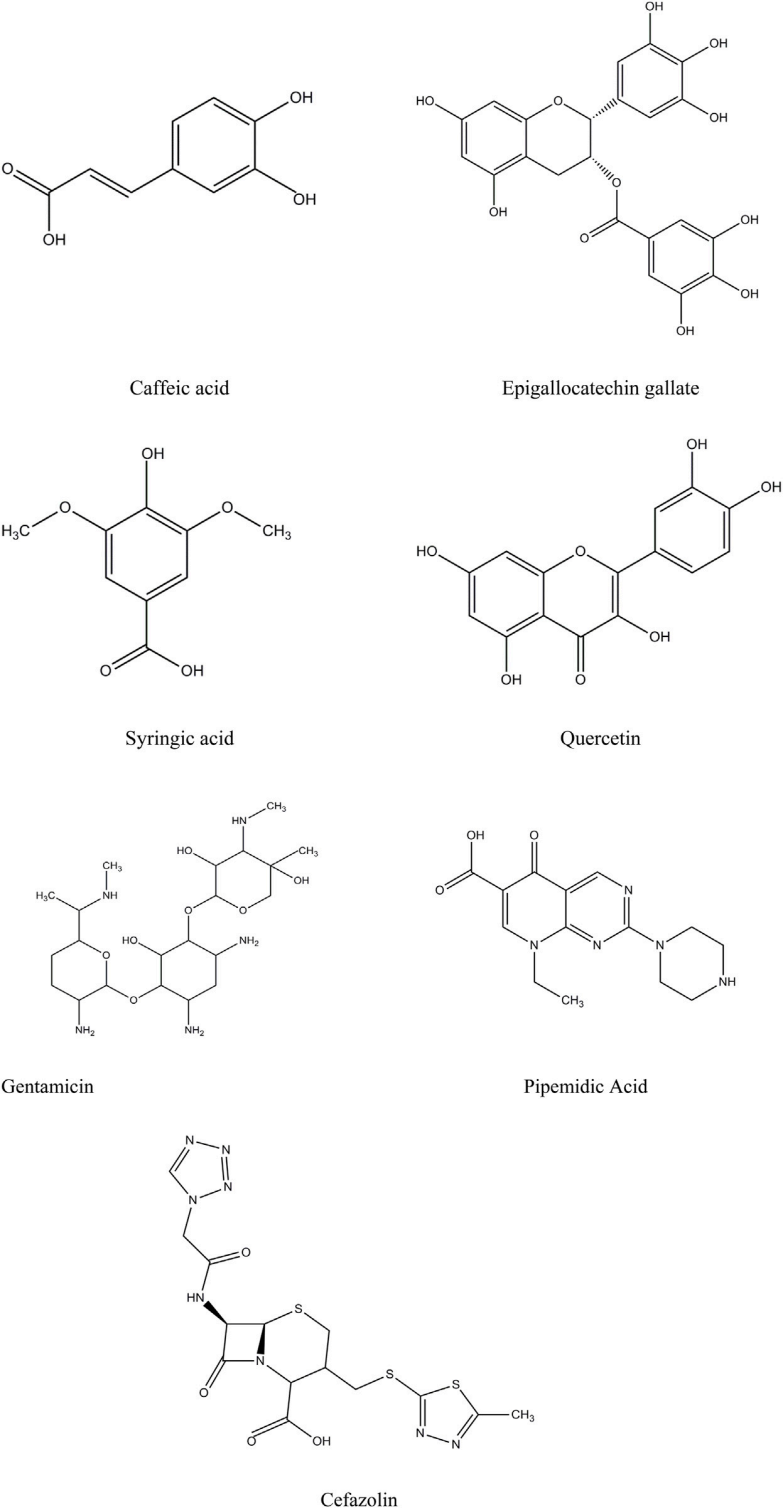
2D chemical structures of the selected ligands and standard antibiotics, generated using ChemDraw Ultra 12.

The selection of candidate ligands for computational analysis was guided by the bioactive compounds identified in C. *sinensis* by HPLC (Figure 1). Their three-dimensional (3D) structures were obtained from the PubChem database (www.pubchem.ncbi.nlm.nih.gov) (Li et al., 2010) that, provides the detailed molecular data required for accurate computational modelling. The 3D structures of the selected bioactive compounds (caffeic acid, EGCG, syringic acid, and quercetin) were verified and optimized for molecular docking and dynamics simulations by energy minimization to reduce steric clashes and improve docking accuracy. Standard antibiotics (gentamicin, pipemidic acid, and cefazolin) (Figure 1), were also included as reference ligands for comparison and underwent the same preparation and optimization process.

#### 2.4.2 Drug-likeness prediction

The drug-likeness and pharmacokinetic properties of the selected compounds and standard antibiotics were evaluated using the Swiss ADME online platform (http://www.swissadme.ch/index.php). The SMILES notations of the compounds were inputted into the tool to analyse their physicochemical properties, Absorption, Distribution, Metabolism, and Excretion (ADME) parameters, and drug-likeness attributes. This analysis ensured that the compounds met Lipinski’s Rule of Five (Lipinski et al., 1997), the Ghose filter (Ghose et al., 1999), and other relevant pharmacokinetic criteria, to determine their suitability as potential drug candidates from a pharmacokinetic and medicinal chemistry perspective. The SwissADME platform results included key parameters, such as molecular weight, lipophilicity (Log P), solubility, and pharmacokinetic properties (e.g., permeability and toxicity predictions) (Boudou et al., 2024a).

#### 2.4.3 Toxicity risk assessment

The toxicity potential of the selected natural compounds and standard antibiotics was assessed using OSIRIS Property Explorer (Chandra et al., 2012). This tool evaluates mutagenic, tumorigenic, irritant, and reproductive toxicity effects. The tool quickly validates chemical structures and predicts their drug-related properties. Toxicity risks were visualized using a color-coded system: red indicates high toxicity risk, while green highlights attributes favourable for drug development (Azad et al., 2018). These results guided the selection of compounds with the most favourable toxicity profiles for further analysis.

#### 2.4.4 Quantum chemical calculations

The quantum chemical properties of the selected ligands (caffeic acid, epigallocatechin gallate (EGCG), syringic acid, and quercetin) and standard antibiotics (gentamicin, pipemidic acid, and cefazolin) were analyzed using Density Functional Theory (DFT). Calculations were performed at the B3LYP-D3BJ/6-31G+* level of theory for neutral and cationic molecules, and the B3LYP-D3BJ/6-31G++* level for anionic molecules, employing the Gaussian 09W software package. The inclusion of Grimme’s D3BJ dispersion correction enhances the accuracy of non-covalent interaction modeling.

The molecular structures of all ligands were fully optimized to their lowest energy conformations. GaussView was utilized for molecular visualization and orbital analysis, facilitating the rendering of molecular structures and the spatial distribution of the frontier molecular orbitals. Key electronic properties, including the Total Energy (E_tot), the energies of the Highest Occupied Molecular Orbital (HOMO) and the Lowest Unoccupied Molecular Orbital (LUMO), the HOMO-LUMO energy gap (ΔE), Dipole Moment (μ), and Molecular Volume (V), were calculated based on the optimized structures. The HOMO-LUMO energy gap (Eg) was determined as the energy difference between these orbitals, providing insights into the electronic stability and reactivity of each compound.

Additionally, the HOMO and LUMO orbitals were visualized to highlight electron-rich and electron-deficient regions, which are critical for understanding chemical interactions and potential binding affinities to target proteins. These quantum chemical parameters contribute to evaluating the electronic features that may influence the bioactivity of the studied compounds.

#### 2.4.5 Selection of target protein and molecular docking

**FIGURE 2:**
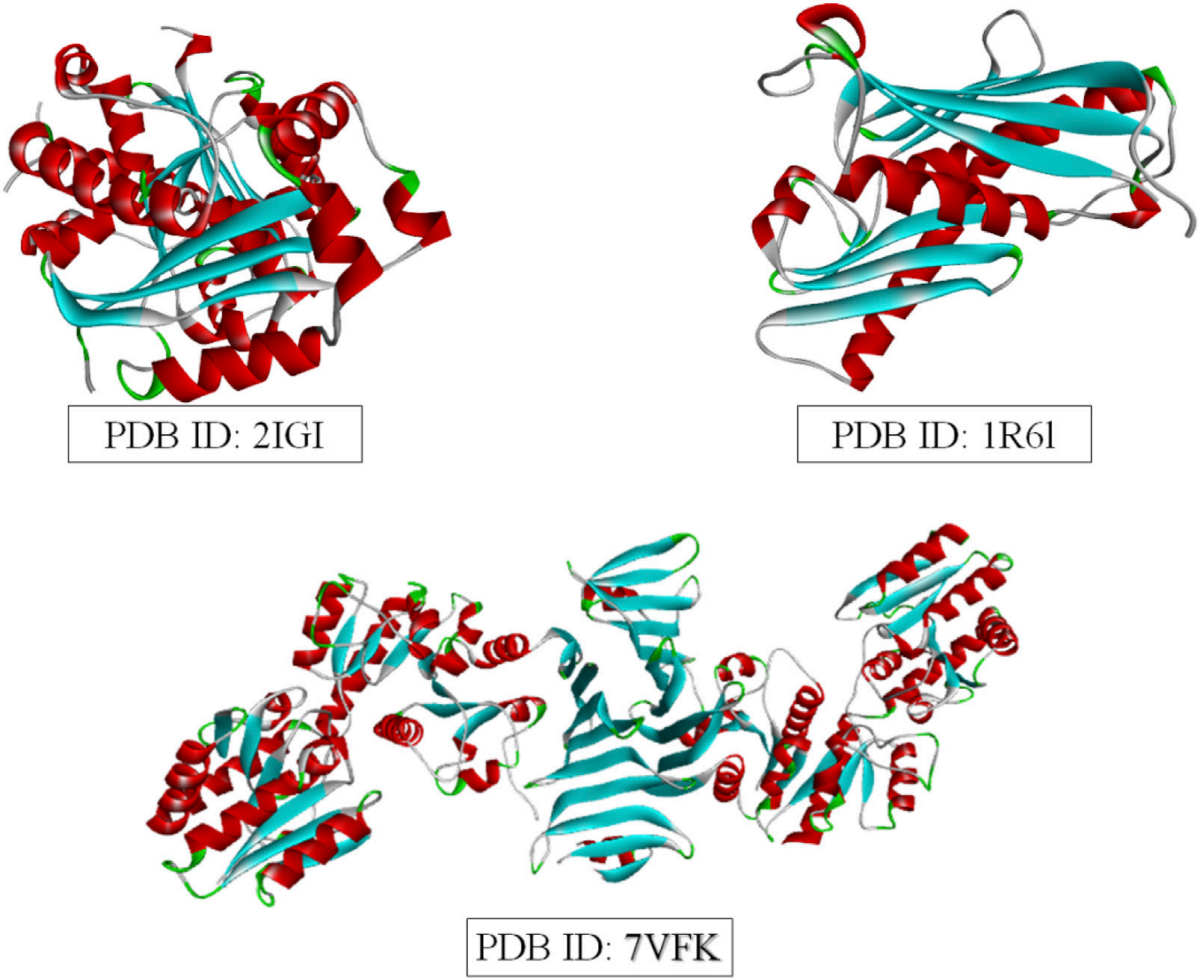
3D Structures of the three target proteins were visualized using BIOVIA Discovery Studio.

Molecular docking was performed to evaluate the binding affinity and interactions of the selected ligands to three bacterial target proteins: *E. coli oligo ribonuclease* (PDB ID: 2IGI), *P. aeruginosa* RNase PH (PDB ID: 1R6L), and the *S. aureus* glycosylation enzyme SdgB (PDB ID: 7VFK) (Figure 2). The protein structures were obtained from the RCSB Protein Data Bank (www.rcsb.org) and prepared for docking studies. To ensure accurate binding site coverage, docking grid box parameters were optimized for each protein, as summarized in Table 1. Ligands were retrieved from PubChem and underwent energy minimization using the UFF force field with the conjugate gradients’ algorithm, which involved 200 optimization steps and a convergence criterion of an energy difference of 0.1 kcal/mol. Molecular docking simulations were carried out using PyRx-Python 0.8 with an exhaustiveness parameter of 8 to ensure a comprehensive exploration of binding poses. The docking results were presented using BIOVIA Discovery Studio Visualizer to examine key protein-ligand interactions, including hydrogen bonds, hydrophobic interactions, and electrostatic contacts between ligands and amino acid residues in the target proteins. The binding affinity of each ligand to the target proteins was quantified using docking scores and free energy of binding.

**TABLE 1:** Docking grid box parameters for the selected bacterial target proteins.

Protein target	PDB ID	Grid box center coordinates (X, Y, Z)	Grid box dimensions (Å)
*E. coli* oligoribonuclease	2IGI	X: 57.1543, Y: 12.1346, Z: 43.8228	X: 51.92, Y: 49.42, Z: 61.29
*P. aeruginosa* RNase PH	1R6L	X: 80.1647, Y: 16.703, Z: −15.6785	X: 43.90, Y: 51.10, Z: 54.78
*S. aureus* SdgB	7VFK	X: 7.8356, Y: −0.0071, Z: 28.142	X: 72.32, Y: 110.95, Z: 123.78

#### 2.4.6 Molecular dynamics simulations

Based on the binding free energy values obtained from the molecular docking analysis, the ligands with the lowest binding free energy, indicative of stronger and more stable interactions with the target proteins, we selected for Molecular dynamics simulations. The simulations were performed using the GROMACS 2023-GPU package and the CHARMM36m force field to validate the docking results and assess the dynamic stability of the ligand-protein complexes. Each selected complex was solvated in a triclinic box using TIP3P water models and neutralized by adding Na^+^ and Cl^−^ ions at a concentration of 150 mM. Energy minimization was carried out to eliminate steric clashes and optimize the system geometry, followed by equilibration under NVT (constant volume and temperature) and NPT (constant pressure and temperature) ensembles for 2 nanoseconds each. The production phase of the molecular dynamic simulation was conducted for 100 nanoseconds at a constant temperature of 300 K and a pressure of 1 bar according to (Boudou et al., 2024b). The molecular dynamics trajectories were analyzed to evaluate the global protein behaviour, local backbone flexibility, and protein-ligand interaction stability over time. The root-mean-square deviation (RMSD), Root-mean-square fluctuation (RMSF), and Radius of gyration (Rg) were computed to confirm the robustness of the docking-predicted binding modes.

## 3 Results and discussion

### 3.1 Phenolic compounds identification

**FIGURE 3:**
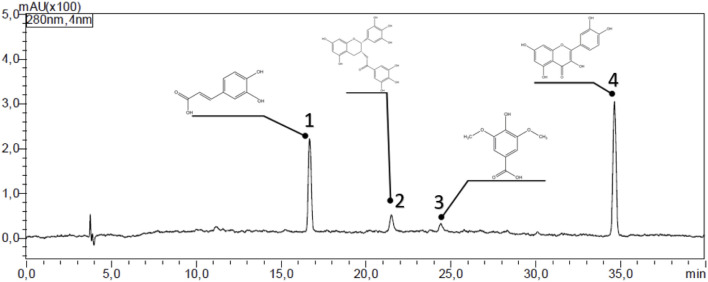
HPLC chromatogram showing the polyphenolic compounds in the *C. sinensis* extract*.* 1: caffeic acid, 2: Epigallocatechin gallate, 3: syringic acid, and 4: quercetin.

The HPLC analysis of the *C. sinensis* methanolic extract (Figure 3; Table 2) revealed a diverse profile of bioactive phenolic compounds. Caffeic acid, EGCG, syringic acid, and quercetin could be separated and identified based on their distinct retention times. Quercetin was the most abundant (15.29% of the extract), followed by caffeic acid (5.52%) EGCG (2.39%), and Syringic acid (1.85%). These compounds exhibited retention times ranging from 16.7 to 34.7 min (Table 2) highlighting the extract-rich and varied phytochemical composition.

**TABLE 2:** Phytochemical profile of the *Camellia sinensis* extract by HPLC.

Peak label	Retention time (min)	Lambda max (nm)	Compound	Conc. (%)	PubChem CID
1	16.7	205/274/321	Caffeic acid	5.52	689,043
2	21.5	205/274/396	Epigallocatechin gallate	2.39	65,064
3	24.4	267/337/380	Syringic acid	1.85	10,742
4	34.7	237/274/357/370/318	Quercetin	15.29	5,280,343

Quercetin, a flavonoid with strong antioxidant properties, has been extensively studied for its role in modulating oxidative stress, inflammation, and microbial resistance (Azeem et al., 2023). Caffeic acid, a hydroxycinnamic acid derivative, has anti-inflammatory, antimicrobial, and antioxidant effects (Li et al., 2016). EGCG, a major catechin in green tea, exhibits potent antioxidant, anticancer, and anti-inflammatory activities (Zhao et al., 2019). Syringic acid, a phenolic acid, has been recognized for its antimicrobial and antioxidant properties (Liu et al., 2020).

These findings are consistent with prior studies that reported a significant abundance of polyphenolic compounds in *C. sinensis* and their diverse pharmacological activities. For instance, Jeszka-Skowron et al. (2015) highlighted the antioxidant properties of these polyphenols, while Chen et al. (2022) demonstrated their potential for managing various chronic diseases through their bioactive mechanisms.

### 3.2 *In vitro* antibacterial activity

**FIGURE 4:**
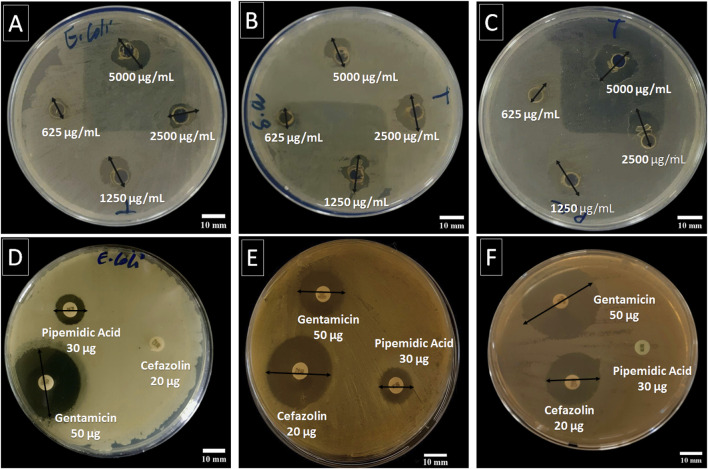
Antibacterial activity of the *C. sinensis* extract at different concentrations (5,000, 2,500, 1,250, and 625 μg/mL) against *E. coli* ATCC 25922 **(A)**, *S. aureus* ATCC 25923 **(B)**, and *P. aeruginosa* ATCC 27853 **(C)** using the agar diffusion method, compared to standard antibiotic (gentamicin 50 μg, pipemidic acid 30 μg, and cefazolin 20 µg) against *E. coli* **(D)**, *S. aureus* **(E)**, and *P. aeruginosa* **(F)**. The two-way arrow represents the inhibition zone diameter.

Table 3; Figure 4 present the inhibition zones (in mm) for the tested bacterial strains exposed to different concentrations of *C. sinensis* extract. At the highest extract concentration (5,000 μg/mL), the largest inhibition zone was observed for *P. aeruginosa* ATCC 27853 (18.65 ± 0.01 mm), followed by *E. coli* ATCC 25922 (16.85 ± 0.10 mm), and *S. aureus* ATCC 25923 (13.74 ± 0.73 mm). As the concentration decreased, inhibition zones were reduced for all strains. At 2,500 μg/mL, *E. coli* and *S. aureus* exhibited similar inhibition zones, while *P. aeruginosa* was still significantly inhibited. At the lowest concentration (625 μg/mL), *S. aureus* had the smallest inhibition zone (8.68 ± 0.20 mm), highlighting its reduced susceptibility compared with the other strains (Figure 1). In summary, the largest zones across all concentrations were observed for *P. aeruginosa,* indicating a higher sensitivity to the extract compared with the other two bacteria. This suggests that the bioactive compounds in the extract have a more potent effect on Gram-negative bacteria, possibly due to the higher permeability of the Gram-negative cell wall to phenolic compounds (Tavares et al., 2020). *S. aureus*, a Gram-positive bacterium, showed the least susceptibility, particularly at lower concentrations, indicating that the extract efficacy varies based on the bacterial structure and resistance mechanisms.

**TABLE 3:** Inhibition zones (in mm) for the tested bacteria exposed to different concentrations of *Camellia sinensis* extract or standard antibiotics.

Plant extract/Antibiotic	Concentration	Inhibition zones (mm)		
		*E. coli ATCC 25922 (Gram-negative)*	*S. aureus ATCC 25923 (Gram-positive)*	*P. aeruginosa ATCC 27853 (Gram-negative)*
*C. sinensis*	5,000 µg/mL	16.85 ± 0.10	13.74 ± 0.73	18.65 ± 0.01
	2,500 µg/mL	11.85 ± 1.02	14.61 ± 0.97	17.04 ± 0.14
	1,250 µg/mL	15.32 ± 0.33	16.59 ± 0.38	17.64 ± 0.38
	630 µg/mL	10.62 ± 0.16	8.68 ± 0.20	12.45 ± 0.43
	312 µg/mL	16.85 ± 0.10	13.74 ± 0.73	18.65 ± 0.01
Gentamicin	50 µg/disc	31.8 ± 0.2	18.9 ± 0.4	35.7 ± 0.0
Pipemidic acid	30 µg/disc	13.8 ± 0.5	14.7 ± 0.4	Nil
Cefazolin	20 µg/disc	nil	28.7 ± 0.8	26.2 ± 1.6

The antibacterial activity of gentamicin, pipemidic acid, and cefazolin was evaluated as benchmarks against *C. sinensis*. Gentamicin showed the strongest inhibition, with inhibition zones of 31.8 ± 0.2 mm for *E. coli*, 18.9 ± 0.4 mm for *S. aureus*, and 35.7 ± 0.0 mm for *P. aeruginosa*. Pipemidic acid exhibited moderate activity against *E. coli* and *S. aureus* (13.9 ± 0.4 mm and 14.6 ± 0.3 mm) but was ineffective against *P. aeruginosa*. Cefazolin was active against *S. aureus* and *P. aeruginosa* (inhibition zones of 28.9 ± 0.8 mm and 26.2 ± 1.6 mm) but showed no activity against *E. coli*. Although *C. sinensis* had lower inhibition zones (e.g.,16.85 ± 0.10 mm for *E. coli* at 5,000 μg/mL), it offers advantages as a natural, sustainable source of bioactive compounds with antioxidant and anti-inflammatory benefits (Awad et al., 2021) and broad-spectrum anti-bacterial activity and at lower risk of inducing bacterial resistance. Therefore, it represents a cost-effective alternative or complement to synthetic antibiotics, particularly against resistant bacterial strains (Veiko et al., 2023).

### 3.3 Drug-likeness prediction

The drug-likeness of *C. sinensis* compounds was predicted using SwissADME (Table 4). Key parameters included molecular weight (MW), number of hydrogen bond acceptors (HBA) and hydrogen bond donors (HBD), molar refractivity (MR), lipophilicity (MLOGP and WLOGP), and compliance with Lipinski’s Rule of Five and the Ghose filter. Caffeic acid (MW: 180.16 g/mol, HBA: 4, HBD: 3) and syringic acid (MW: 198.17 g/mol, HBA: 5, HBD: 2) fully complied with both Lipinski’s rule and the Ghose filter, indicating favorable drug-like properties. Quercetin (MW: 302.24 g/mol, HBA: 7, HBD: 5) also showed no violations, making it a promising candidate. Conversely, EGCG (MW: 458.37 g/mol, HBA: 11, HBD: 4) violated Lipinski’s rule due to exceeding HBA and HBD thresholds but still met the Ghose filter criteria. In comparison, gentamicin (MW: 477.60 g/mol) and cefazolin (MW: 454.51 g/mol) had higher MW values and multiple violations of drug-likeness rules. Gentamicin exhibited two Lipinski violations (NorO >10, NHorOH >5) and was disqualified by the Ghose filter due to WLOGP < −0.4 and exceeding 70 atoms. Cefazolin violated only one, Lipinski’s rule but did not pass the Ghose filter (WLOGP < −0.4). Like quercetin pipemidic acid (MW: 303.32 g/mol), showed strong drug-likeness (Lipinski’s rule violations), but did not pass the Ghose filter (WLOGP < −0.4).

**TABLE 4:** Drug-likeness prediction of the polyphenolic compounds identified in *Camellia sinensis* and standard antibiotics using SwissADME.

Plant source	Ligand (formula)	MW (g/mol)	HBA number	HBD number	Molar refractivity	MLOGP	WLOGP	Lipinski rule	Ghose filter
Caffeic acid	C9H8O4	180.16	4	3	47.16	0.70	1.09	Yes; 0 violation	Yes
Epigallocatechin gallate	C22H18O11	458.37	11	4	112.06	−0.44	1.91	No; 2 violations: NorO>10, NHorOH>5	Yes
Syringic acid	C9H10O5	198.17	5	2	48.41	0.49	1.11	Yes	Yes
Quercetin	C15H10O7	302.24	7	5	78.03	−0.56	1.99	Yes; 0 violation	Yes
Gentamicin	C21H43N5O7	477.60	12	8	118.31	−2.92	−3.33	No; 2 violations: NorO>10, NHorOH>5	No; 2 violations: WLOGP < −0.4, #atoms>70
Cefazolin	C14H14N8O4S3	454.51	9	2	106.69	0.08	−1.02	Yes; 1 violation: NorO>10	No; 1 violation: WLOGP < −0.4
Pipemidic Acid	C14H17N5O3	303.32	6	2	88.19	0.04	−0.84	Yes; 0 violation	No; 1 violation: WLOGP < −0.4

**FIGURE 5:**
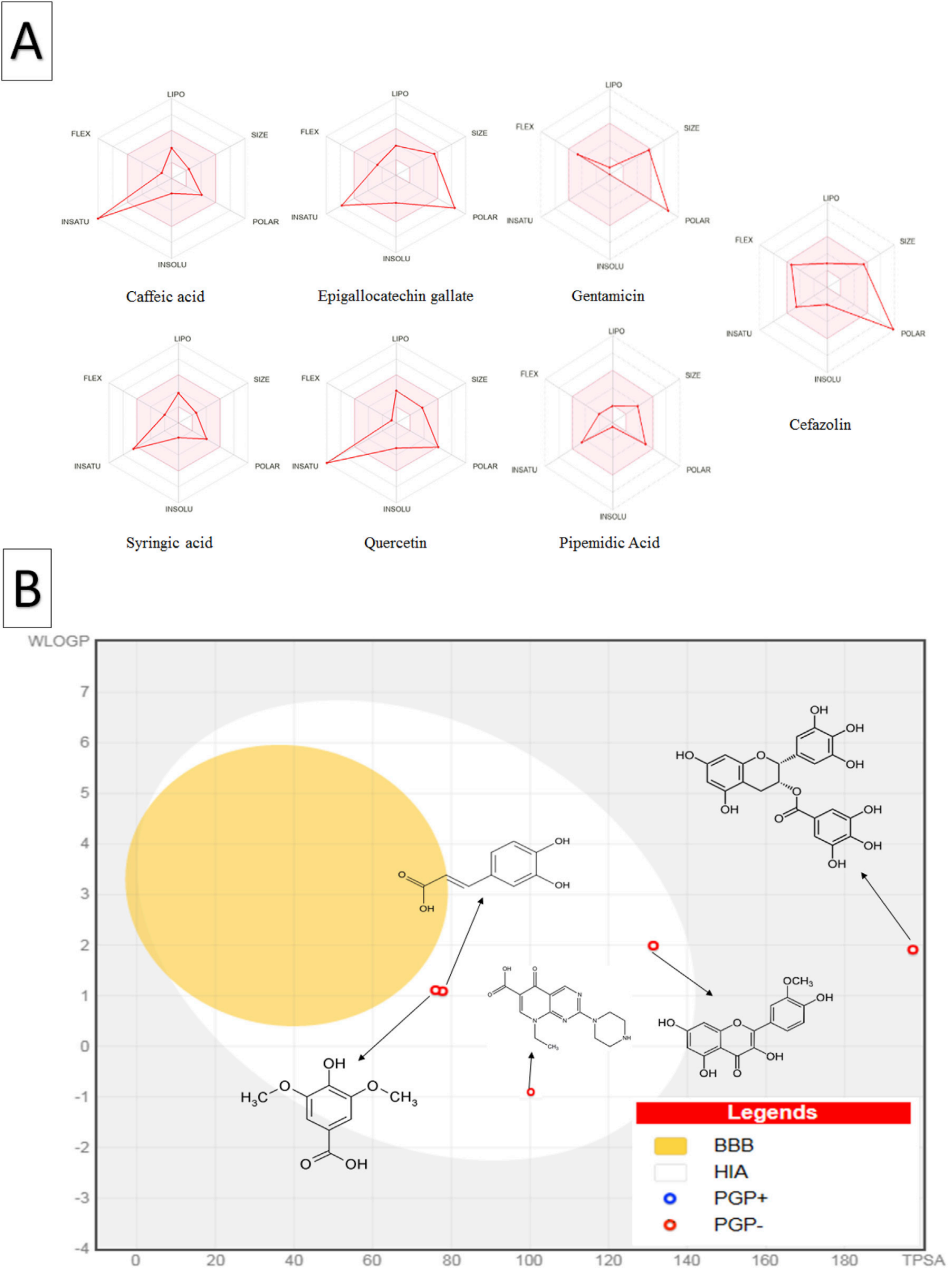
Oral bioavailability and blood-brain barrier absorption analysis of *C. sinensis* polyphenolic compounds and standard antibiotics using the SwissADME database **(A)** The coloured zone in the oral bioavailability radar plot represents the optimal physicochemical space for oral bioavailability, the red line depicts the properties of the compounds **(B)** The BOILED-Egg plot shows the predicted human blood-brain barrier absorption; the red dots indicate that the molecule is not expected to be effluxed from the central nervous system by p-glycoproteins. Abbreviations: LIPO (lipophilicity), POLAR (polarity), INSOLU (insolubility), INSATU (unsaturation), FLEX (flexibility), BBB (blood-brain barrier), HIA (human intestinal absorption), PGP+ (p-glycoprotein substrate), and PGP- (non-p-glycoprotein substrate).

The bioavailability radar plot (Figure 5A) showed that caffeic acid, syringic acid, and quercetin aligned well with the INSATU zone, indicating optimal drug-like properties, while EGCG displayed deviations toward the POLAR region, reflecting its lower polarity. In the BOILED-Egg plot analysis (Figure 5B), caffeic acid, syringic acid and quercetin were within the white region of the egg, suggesting high human intestinal absorption. Conversely, EGCG was outside this region, indicating lower oral bioavailability. These findings are consistent with previous studies (e.g., Belakredar et al., 2024), highlighting that caffeic acid, syringic acid, and quercetin a promising candidate for drug development based on their favorable physicochemical and drug-likeness profiles.

### 3.4 Toxicity risk assessment

The toxicity risk of the *C. sinensis* compounds identified in this study was predicted using the OSIRIS Property Explorer (Table 5). Caffeic acid and EGCG exhibited generally favourable safety profiles, with green indicators across most categories. However, caffeic acid had a red warning concerning reproductive effects. Conversely, quercetin displayed mutagenic and tumorigenic risks, and syringic acid was marked as mutagenic. Despite quercetin’ recognized beneficial properties, its potential mutagenic and tumorigenic effects have been documented in previous studies, urging caution when considering its use for therapeutic purposes (Kim et al., 2019). All the tested antibiotics (gentamicin, cefazolin, and pipemidic acid), displayed green indicators across all toxicity categories.

**TABLE 5:** OSIRIS Property Explorer prediction of the toxicity risk of *Camellia sinensis* phytochemical and tested antibiotics.

Compound	Mutagenic	Tumorigenic	Irritant	Reproductive effect
Caffeic acid	Green	Red	Green	Red
Epigallocatechin gallate	Green	Green	Green	Green
Syringic acid	Red	Green	Green	Green
Quercetin	Red	Red	Green	Green
Gentamicin	Green	Green	Green	Green
Cefazolin	Green	Green	Green	Green
Pipemidic Acid	Green	Green	Green	Green

### 3.5 Density Functional Theory calculation

**FIGURE 6:**
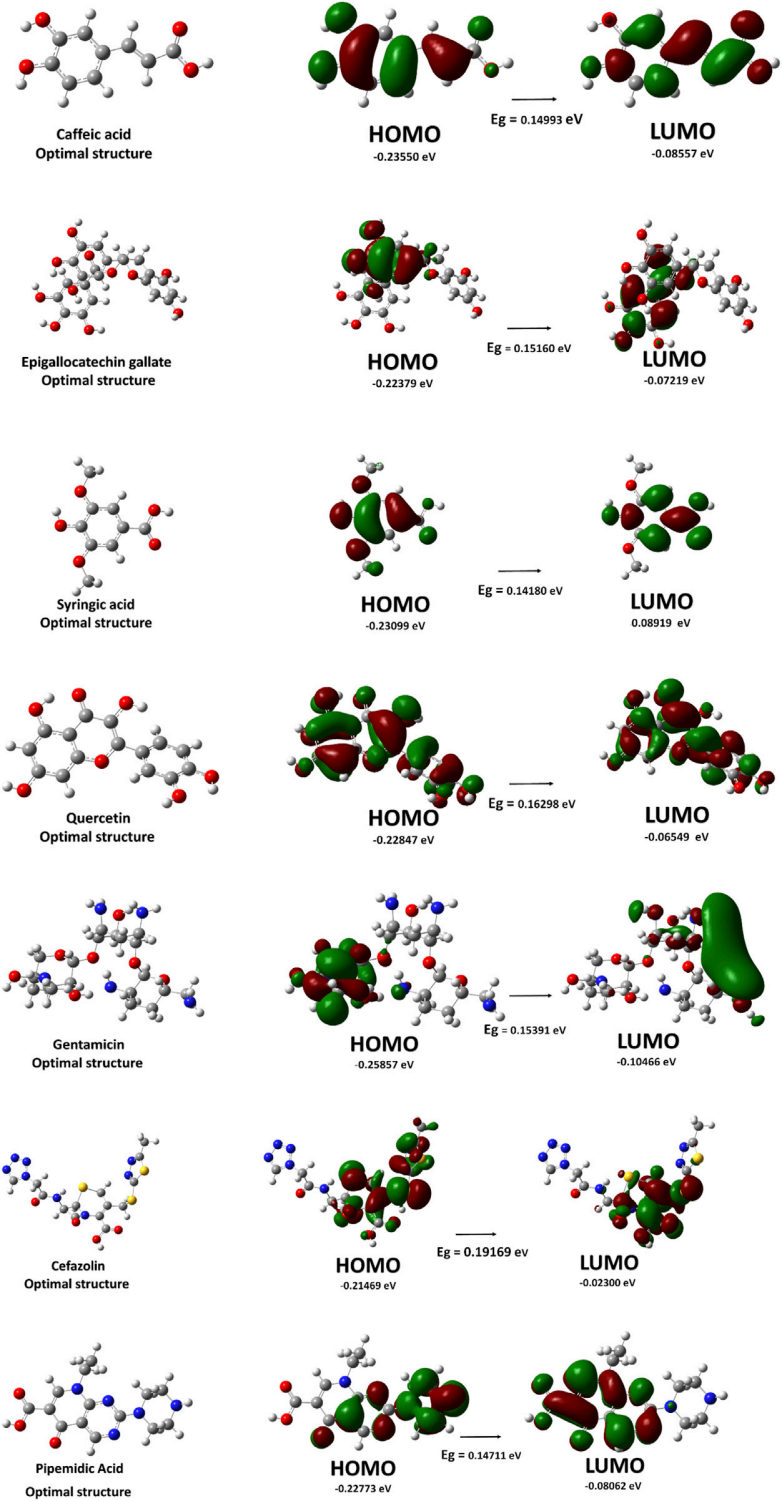
Optimized molecular structures and frontier molecular orbitals (HOMO and LUMO), along with the energy gap (E.g.,) values, of selected *C. sinensis* compounds and reference antibiotics.

Density Functional Theory (DFT) calculations were performed to analyze the electronic properties, stability, and molecular reactivity of selected compounds. The computed parameters include total energy (ΔE), frontier molecular orbital energies (EHOMO and ELUMO), energy gap (ΔE), dipole moment (μ), and molar volume (V), as presented in Table 6; Figure 6.

**TABLE 6:** Electronic properties of selected compounds from *Camellia sinensis* and reference antibiotics.

Compound	Etot	EHOMO (eV)	ELUMO (eV)	ΔE (eV)	μ (D)	V (cm^3^/mol)
Caffeic acid	−648.54	−0.23550	−0.08557	0.14993	5.47	150.13
Epigallocatechin gallate	−1,676.31	−0.22379	−0.07219	0.15160	3.78	255.22
Syringic acid	−1,103.96	−0.23099	−0.08919	0.14180	5.87	251.87
Quercetin	−724.93	−0.22847	−0.06549	0.16298	3.07	165.14
Gentamicin	−2,474.92	−0.25857	−0.10466	0.15391	8.30	378.75
Cefazolin	−1,547.52	−0.21469	−0.02300	0.19169	4.11	398.00
Pipemidic Acid	−1,042.98	−0.22773	−0.08062	0.14711	8.05	251.93

The energy of the highest occupied molecular orbital (EHOMO) and the lowest unoccupied molecular orbital (ELUMO) provide insight into the electron-donating and accepting capabilities of the molecules. Cefazolin exhibited the lowest EHOMO (−0.25857 eV), indicating its relatively lower electron-donating ability compared to the other compounds. Conversely, gentamicin displayed the highest ELUMO (−0.02300 eV), suggesting reduced electron affinity and a lower tendency for electrophilic interactions.

The HOMO-LUMO energy gap (ΔE) is a crucial parameter for understanding molecular stability and chemical reactivity. Among the studied compounds, gentamicin exhibited the highest energy gap (0.19169 eV), indicating greater stability and lower reactivity. On the other hand, quercetin presented the lowest energy gap (0.14180 eV), implying increased reactivity and potential interactions with biological targets.

The dipole moment (μ) reflects the polarity of a molecule, which is critical for solubility and intermolecular interactions. Cefazolin and pipemidic demonstrated the highest dipole moments (8.30 D and 8.05 D, respectively), suggesting strong intermolecular interactions and potential for hydrogen bonding. In contrast, syringic acid had the lowest dipole moment (3.07 D), indicating lower polarity and possible reduced solubility in polar environments.

The estimated molar volumes (V) provide insight into the molecular size and potential steric effects. Gentamicin exhibited the highest molar volume (398.00 cm^3^/mol), which may impact its diffusion and bioavailability. Conversely, syringic acid showed the lowest molar volume (165.14 cm^3^/mol), suggesting its smaller molecular size and higher potential for cellular penetration.

The DFT calculations reveal significant variations in electronic properties among the selected compounds. Quercetin, with the smallest HOMO-LUMO gap, appears to be the most reactive, whereas gentamicin is the most stable. The dipole moment and molar volume further contribute to understanding the solubility and bioavailability of these molecules, aiding in the rational selection of candidates for further experimental validation. Additionally, molecular docking results highlight EGCG as the most promising candidate due to its superior binding affinities across all bacterial targets, outperforming standard antibiotics. Quercetin also demonstrated notable binding potential, suggesting its role as a viable antimicrobial agent with additional health benefits. These findings underscore the potential of plant-derived polyphenols as complementary or alternative antimicrobial agents, warranting further *in vitro* and *in vivo* investigations.

### 3.6 Molecular docking

The molecular docking analysis (Table 7) assessed the binding affinities and interactions of the natural ligands and standard antibiotics against three bacterial target proteins: *E. coli* oligo-ribonuclease (PDB ID: 2IGI), *P. aeruginosa* RNase PH (PDB ID: 1R6L), and *S. aureus* SdgB (PDB ID: 7VFK). The data revealed differences in the binding potential of plant-derived compounds compared with the tested antibiotics, highlighting their potential as alternative antimicrobial agents with additional therapeutic benefits.

**TABLE 7:** Binding affinity and molecular interactions of three target bacterial proteins with the selected *C.* sinensis phenolic compounds and standard antibiotics.

Proteins	Ligands (PubChem CID)	Binding free energy (kcal/mol)	Binding residue
*E. coli* oligo-ribonuclease (PDB ID: 2IGI)	Caffeic acid (CID: 689,043)	−6.5	Glu B.13, His B.15, Met B.14
	Epigallocatechin gallate (CID: 65,064)	−9.8	Asp A.111, Gln A.110, Leu A.12, His A.157, Trp A.128
	Syringic acid (CID: 10,742)	−5.9	Arg A.112, Glu A.123, Leu B.17, Phe A.126, Phe A.116, Tyr A.130, Tyr A.128
	Quercetin (CID: 5,280,343)	−8.7	Asp B.11, Asp B.162, Glu B.165, His B.157
	Gentamicin (CID: 72,396)	−8.2	Arg A.122, Asp B.11, Asp B.162, Ser B.107
	Pipemidic acid (CID:4831)	−7.7	Leu B.12, Glu B.13, Met B.14, Asn B.61, His B.157, Leu B.17, Trp B.60
	Cefazolin (CID: 33,255)	−8.6	Asp A.11, Glu B.13, Leu B.17, Phe B.116
*P. aeruginosa* RNase PH(PDB ID: 1R6l)	Caffeic acid (CID: 689,043)	−5.7	Glu A:73, Tyr A:178, Arg A:87, Ala A:122
	Epigallocatechin gallate (CID: 65,064)	−7.8	Arg A:74, Mse A:66, Tyr A:178, Arg A:87
	Syringic acid (CID: 10,742)	−5.0	Asn A:189, Asp A:187, Asp A:181, Glu A:90
	Quercetin (CID: 5,280,343)	−6.9	Ala A:122, Thr A:71, Tyr A:178
	Gentamicin (CID: 72,396)	−6.1	Gln A:202, Thr A:204, Glu A:206, Asp A:187, Glu A.90, Arg A:87
	Pipemidic acid (CID:4831)	−6.9	Gln A:76, Glu A:78, Arg A:81, Mse A:66
	Cefazolin (CID: 33,255)	−7.0	Ala A:113, Ile A:167, Thr A:186, Glu A:135, Ala A:208, Pro A.172, Tyr A:168
*S. aureus* SdgB (PDB ID: 7VFK)	Caffeic acid (CID: 689,043)	−6.1	Asn A: 165, Tyr A:164, Tyr B:191, Glu B:173
	Epigallocatechin gallate (CID: 65,064)	−8.3	Tyr B:191, Gly A:168, Tyr A:164, Glu B:173
	Syringic acid (CID: 10,742)	−5.6	Asp B:154, Arg B:133
	Quercetin (CID: 5,280,343)	−7.9	Tyr A:191, Glu A:173, Leu B:146, Tyr B:164, Asn B:165
	Gentamicin (CID: 72,396)	−6.6	Lys B:195, Gly A:168
	Pipemidic acid (CID:4831)	−7.5	Leu A:146, Tyr B:191, Ser A:149, Thr B:170
	Cefazolin (CID: 33,255)	−7.1	Leu B:386, Ser B:379, Arg B:329, Phe B:408, His B:246, Val B:308

Specifically, EGCG exhibited the strongest binding affinity (−9.8 kcal/mol) to *E. coli* oligo-ribonuclease (PDB ID: 2IGI) among all tested compounds. This high binding strength is due to its interactions with critical residues, including Asp A:111, Gln A:110, and Trp A:128. EGCG’s potent binding affinity, along with its well-known antioxidant and anti-inflammatory properties, makes it a promising candidate for managing bacterial infections and mitigating the oxidative stress associated with inflammation. Quercetin also had a significant binding affinity (−8.7 kcal/mol) to *E. coli* oligo-ribonuclease, forming hydrogen bonds with Asp B:11 and His B:157. Quercetin has anti-inflammatory and immune-boosting effects and additional health benefits, potentially reducing the side effects associated with antibiotic therapies. Among the standard antibiotics, cefazolin showed a similar binding affinity (−8.6 kcal/mol) followed by, gentamicin (8.2 kcal/mol). Plant-derived Caffeic acid (−6.5 kcal/mol) and Syringic acid (−5.9 kcal/mol) displayed weaker binding, but they may still offer synergistic antioxidant benefits when used in combination therapies.

Similarly, EGCG showed the highest binding affinity --7.8 kcal/mol) for *P. aeruginosa* RNase PH (PDB ID: 1R6), interacting with the key residues Arg A:74 and Tyr A:178. Among the antibiotics, cefazolin displayed a binding affinity of −7.0 kcal/mol, followed closely by pipemidic acid (−6.9 kcal/mol), while gentamicin had a lower affinity (−6.1 kcal/mol). Quercetin binding affinity was in the same range (−6.9 kcal/mol). Quercetin’s role in modulating inflammatory pathways and supporting overall cellular health further emphasizes its therapeutic potential. On the other hand, caffeic acid (−5.7 kcal/mol) and syringic acid (−5.0 kcal/mol) showed relatively weak interactions, although their antibacterial properties Could provide complementary benefits (Liu et al., 2020).

Also for *S. aureus* SdgB (PDB ID: 7VFK), EGCG was the top-performing ligand (−8.3 kcal/mol) interacting with Tyr B:191 and Glu B:173, followed by quercetin (−7.9 kcal/mol), pipemidic acid (−7.5 kcal/mol), cefazolin (−7.1 kcal/mol) and gentamicin (−6.6 kcal/mol) exhibited moderate binding affinities Caffeic acid (−6.1 kcal/mol) and syringic acid (−5.6 kcal/mol) showed the lowest binding affinities, reflecting limited interaction potential with this protein.

**FIGURE 7:**
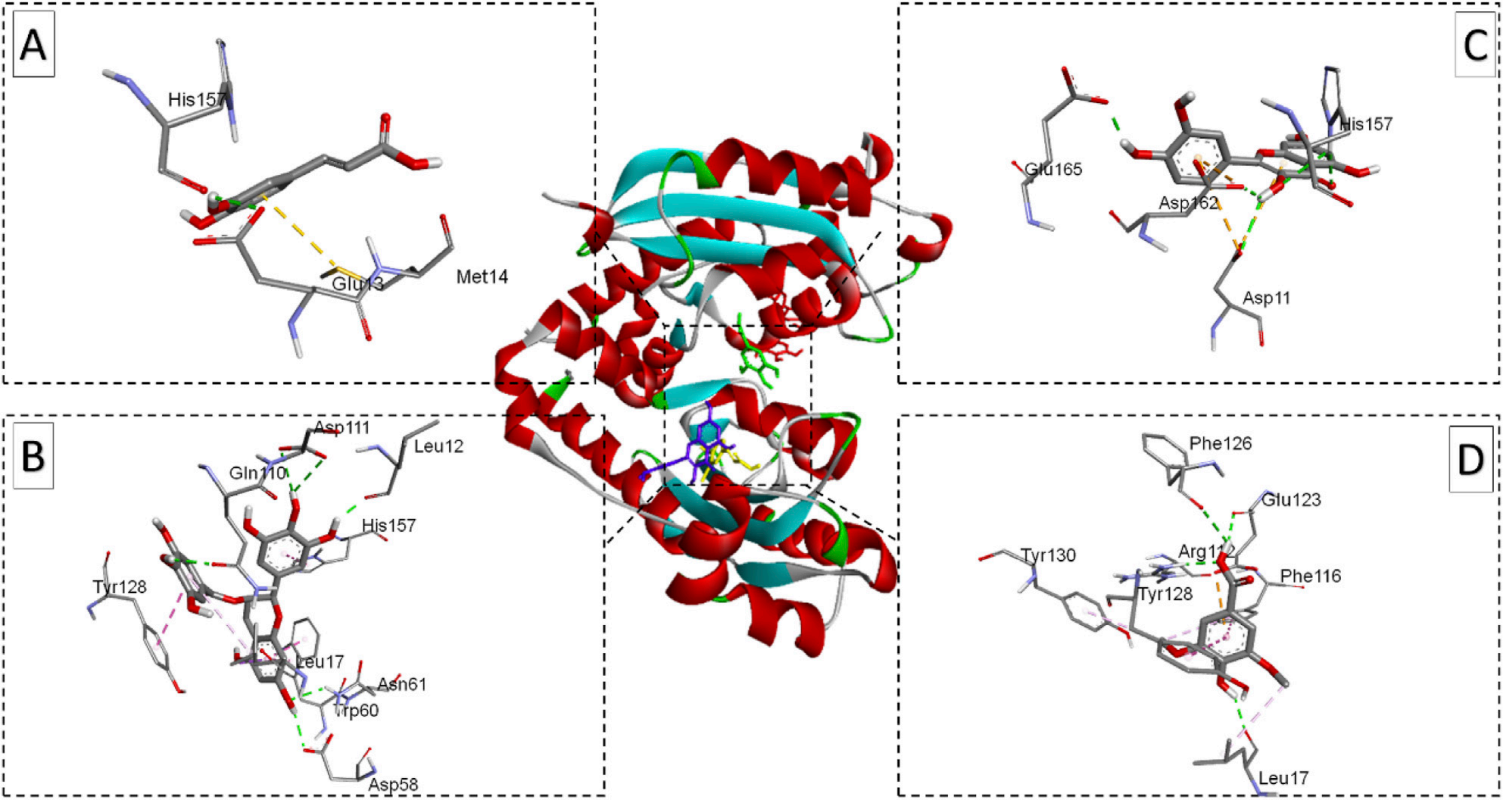
Ribbon views of the target protein (*E. coli* oligo-ribonuclease; PDB ID: 2IGI) and details of the active site (inset) highlighting the enzyme-ligand interactions with the four phenolic compounds. 2IGI-Caffeic acid complex **(A)**, 2IGI-Epigallocatechin gallate complex **(B)**,2IGI-Syringic acid complex **(C)**, and 2IGI-Quercetin complex **(D)**.

**FIGURE 8:**
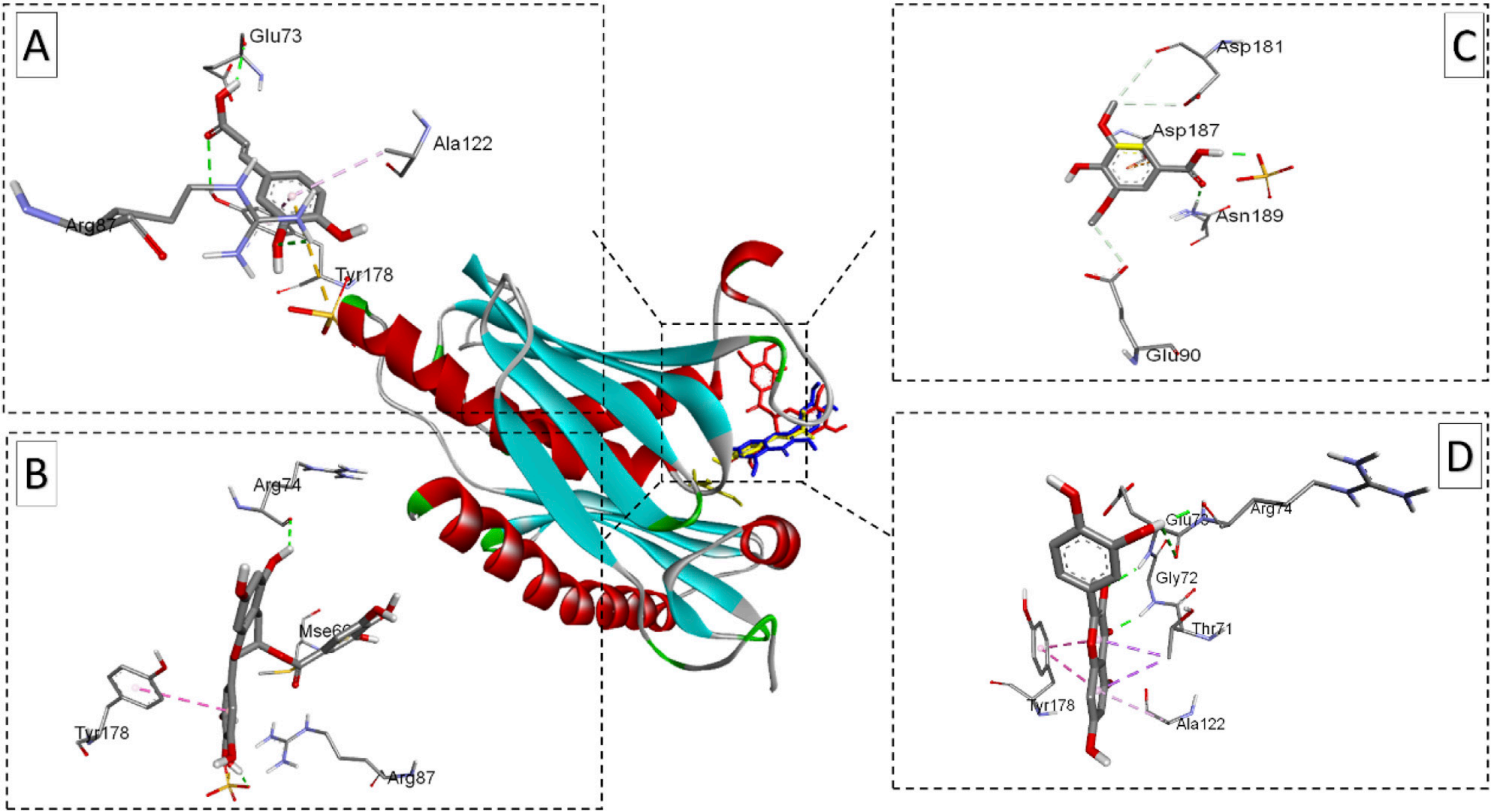
Ribbon views of the target protein (*P. aeruginosa* RNase PH PDB ID: 1R6l) and details of the active site (inset), highlighting the enzyme-ligand interactions with the four phenolic compounds. 1R6l-Caffeic acid complex **(A)**, 1R6l-Epigallocatechin gallate complex **(B)**,1R6l-Syringic acid complex **(C)**, and 1R6l-Quercetin complex **(D)**.

**FIGURE 9:**
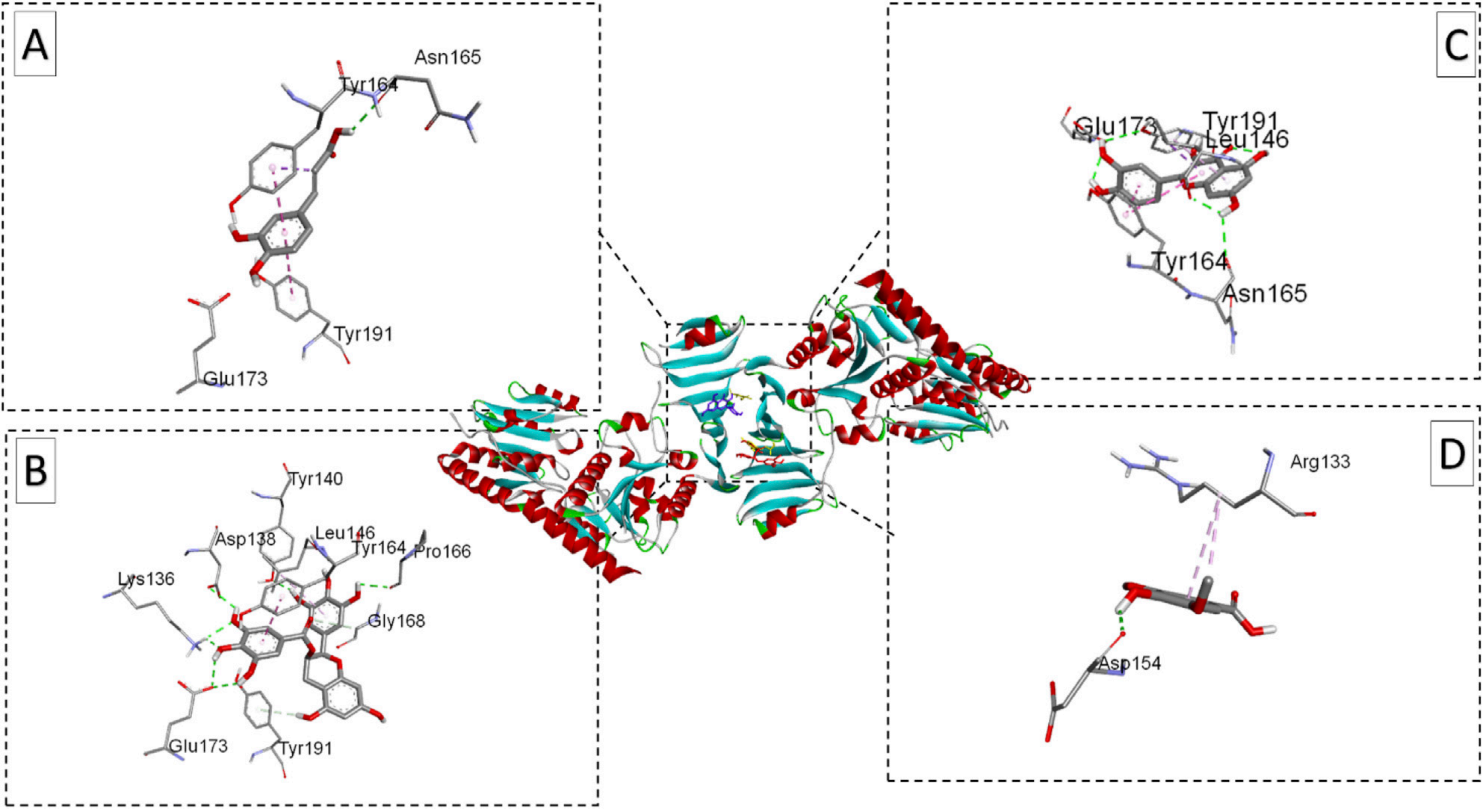
Ribbon views of the target protein (*S. aureus* SdgB; PDB ID: 4ZP4) and details of the active site (inset), highlighting the enzyme-ligand interactions with the four phenolic compounds. 4ZP4-Caffeic acid complex **(A)**, 4ZP4-Epigallocatechin gallate complex **(B)**,4ZP4-Syringic acid complex **(C)**, and 4ZP4-Quercetin complex **(D)**.

The visualizations of these interactions (Figures 7–9), using BIOVIA Discovery Studio Visualizer, offered detailed insights into the binding modes and potential antimicrobial efficacy of the identified phytochemicals. In conclusion, EGCG consistently demonstrated superior binding affinities for all three bacterial target proteins, outperforming standard antibiotics and other plant-derived compounds. Besides its strong binding potential, EGCG is widely recognized for its ability to scavenge free radicals, protect cellular structures, and inhibit the growth of different pathogens, making it a multi-functional therapeutic agent. Quercetin, which exhibited notable binding potential, particularly against *E. coli* oligo-ribonuclease and *S. aureus* SdgB, also contributes to vascular-to-vascular health and immune regulation. The antibiotics Cefazolin and pipemidic acid also performed well showing moderate binding affinities across all three targets (Guilhelmelli et al., 2013).

### 3.7 Molecular dynamics modelling

**FIGURE 10:**
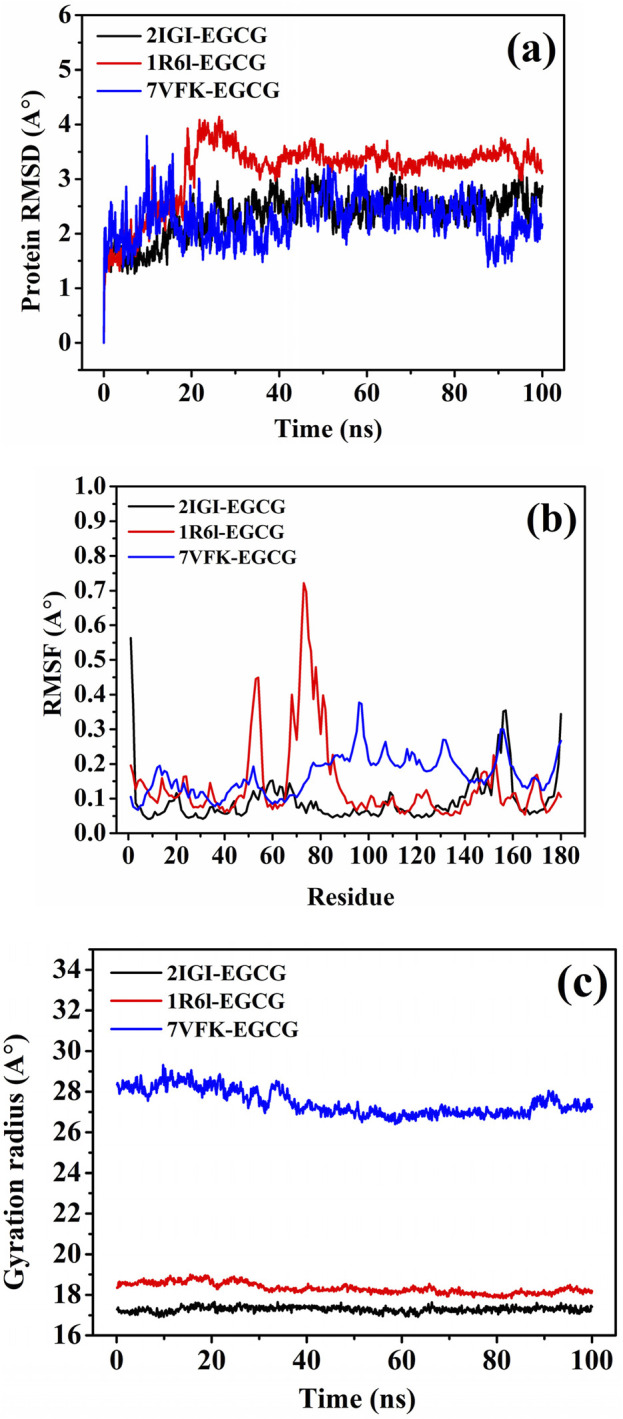
Molecular dynamics analysis of the target proteins *E. coli* oligoribonuclease (2IGI), *P. aeruginosa* RNase PH (1R6L), and *S. aureus* SdgB (7VFK) complexed with EGCG **(A)** Root mean square deviation (RMSD) **(B)** Root mean square fluctuation (RMSF), and **(C)** Radius of gyration (Rg) trajectories.

The RMSD trajectories (Figure 10A) revealed the dynamic stability of the ligand-protein complexes over a 100 ns simulation. The EGCG complexes with 2IGI and 7VFK exhibited lower and more stable RMSD values (∼2.5–3.0 Å), indicating strong and consistent interactions. Conversely, the 1R6L-EGCG complex showed slightly higher RMSD values (∼3.5 Å), reflecting moderate stability. These observations correlate with the molecular docking data, where EGCG showed the strongest binding affinity for 2IGI and 7VFK, suggesting that its higher binding affinity contributes to enhancing the complex stability. These results are in line with previous studies indicating that EGCG’ ability to stabilize elastase binding sites through strong, interactions enhance its therapeutic potential against pathogenic *P. aeruginosa* strains (Najarzadeh et al., 2019).

The RMSF flexibility analysis at the residue level (Figure 10B) indicated that regions involved in docking interactions exhibited distinct fluctuation patterns. For example, residues Asp A:111, Gln A:110, and His A:157 in 2IGI, which are key docking interaction sites for EGCG, showed reduced flexibility, consistent with strong binding. Similarly, in 7VFK, residues Tyr B:191 and Glu B:173, critical for EGCG binding, exhibited lower RMSF values, indicating restricted movement due to stable interactions. For 1R6L, the moderate RMSF values of Arg A:74 and Tyr A:178, which are involved in EGCG binding, are in line with the observed higher RMSD, suggesting less rigid binding. These observations are consistent with studies showing that reduced RMSF values in key binding residues enhance stability and binding efficiency, supporting caffeic acid and EGCG’s favourable binding profiles (Qi et al., 2023).

The Rg computation (Figure 10C) provided additional insights into the compactness of the ligand-protein complexes during the molecular dynamic’s simulations. The Rg values for the 2IGI-EGCG and 1R6L-EGCG complexes remained stable and lower (∼18–20 Å and ∼23–25 Å, respectively), indicating minimal structural fluctuations and greater compactness. Conversely, the 7VFK-EGCG complex exhibited slightly higher and more variable Rg values (∼27–29 Å), reflecting moderate structural flexibility. These results further support the docking data and RMSD findings, highlighting the consistent compactness and stability of the 2IGI-EGCG and 1R6L-EGCG complexes compared with the more dynamic behaviour of the 7VFK-EGCG complex.

The molecular docking and molecular dynamics simulation results collectively highlight the superior performance of EGCG in complex stabilization. Similarly, Angelini, (2024) reported that EGCG’s ability to form multiple hydrogen bonds enhances its affinity and binding stability with penicillin-binding proteins. Overall, the molecular docking and molecular dynamics analyses underscore EGCG as an effective antimicrobial agent, with robust binding and dynamic stability across the tested target proteins. These findings provide a foundation for the experimental validation and optimization of these compounds.

## 4 Conclusion

This study uses experimental and computational methods to evaluate the antibacterial potential of *C. sinensis* leaf extract. The methanolic extract was prepared by maceration and analyzed using HPLC. Key phenolic compounds were identified: quercetin (15.29 mg/g), caffeic acid (10.32 mg/g), EGCG (8.74 mg/g), and syringic acid (6.21 mg/g). *In vitro* antibacterial assays showed significant activity, with inhibition zones ranging from 10.62 mm for *E. coli* to 18.65 mm for *S. aureus*, comparable to gentamicin (19.42 mm). Computational analyses, including drug-likeness predictions, molecular docking, and molecular dynamics simulations, showed strong binding affinities for EGCG (−9.8 kcal/mol) and quercetin (−8.7 kcal/mol) with bacterial target proteins. Molecular dynamics simulations confirmed the stability of EGCG in complex with bacterial targets, with RMSD values of 2.5–3.0 Å, reduced RMSF at key residues, and stable Rg (∼18–20 Å). These results highlight the therapeutic potential of *C. sinensis* polyphenols, especially EGCG, as natural antimicrobial agents with additional antioxidant and anti-inflammatory benefits. Although their inhibition zones are slightly smaller than those of standard antibiotics, their natural origin and lower resistance risk make them promising alternatives. Future research will focus on *in vivo* validation, optimization of structure-activity relationships, and clinical trials to fully explore their therapeutic potential.

## Data Availability

The original contributions presented in the study are included in the article/supplementary material, further inquiries can be directed to the corresponding author.

## References

[B1] Angelini P. (2024). Plant-derived antimicrobials and their crucial role in combating antimicrobial resistance. Antibiotics 13 (8), 746. 10.3390/antibiotics13080746 39200046 PMC11350763

[B2] Awad A. M. Kumar P. Ismail-Fintry M. R. Jusoh S. Ab Aziz M. F. Sazili A. Q. (2021). Green extraction of bioactive compounds from plant biomass and their application in meat as natural antioxidant. Antioxidants 10 (9), 1465. 10.3390/antiox10091465 34573097 PMC8466011

[B3] Azad I. Nasibullah M. Khan T. Hassan F. Akhter Y. (2018). Exploring the novel heterocyclic derivatives as lead molecules for design and development of potent anticancer agents. J. Mol. Graph. Model. 81, 211–228. 10.1016/j.jmgm.2018.02.013 29609141

[B4] Azeem M. Hanif M. Mahmood K. Ameer N. Chughtai F. R. S. Abid U. (2023). An insight into anticancer, antioxidant, antimicrobial, antidiabetic and anti-inflammatory effects of quercetin: a review. Polym. Bull. 80, 241–262. 10.1007/s00289-022-04091-8 PMC8800825 35125574

[B5] Belakredar A. Boudou F. Abdelghani S. (2024). Exploring the anti-inflammatory potential of phytochemicals from *Anvillea radiata*: *in vitro* assay, molecular docking, and molecular dynamics simulations. Adv. Res. Life Sci. 8 (1), 1–14. 10.2478/arls-2024-0001

[B6] Boudou F. Belakredar A. Berkane A. Keziz A. Alsaeedi H. Cornu D. (2024a). Phytochemical profiling and *in silico* evaluation of Artemisia absinthium compounds targeting Leishmania N-myristoyltransferase: molecular docking, drug-likeness, and toxicity analyses. Front. Chem. 12, 1508603. 10.3389/fchem.2024.1508603 39669181 PMC11635459

[B7] Boudou F. Guendouzi A. Belkredar A. Rasheed M. (2024b). An integrated investigation into the antibacterial and antioxidant properties of propolis against *Escherichia coli* cect 515: a dual *in vitro* and *in silico* analysis. Not. Sci. Biol. 16 (2), 13837. 10.55779/nsb16211837

[B8] Chandra S. Chatterjee P. Dey P. Bhattacharya S. (2012). Evaluation of *in vitro* anti-inflammatory activity of coffee against the denaturation of protein. Asian Pac. J. Trop. Biomed. 2 (1), S178–S180. 10.1016/S2221-1691(12)60154-3

[B9] Chen Y. H. Zhang Y. H. Chen G. S. Yin J. F. Chen J. X. Wang F. (2022). Effects of phenolic acids and quercetin-3-O-rutinoside on the bitterness and astringency of green tea infusion. npj Sci. Food 6 (1), 8. 10.1038/s41538-022-00124-8 35087059 PMC8795203

[B10] Ghose A. K. Viswanadhan V. N. Wendoloski J. J. (1999). A knowledge-based approach in designing combinatorial or medicinal chemistry libraries for drug discovery: 1. A qualitative and quantitative characterization of known drug databases. J. Comb. Chem. 1 (1), 55–68. 10.1021/cc9800071 10746014

[B11] Guilhelmelli F. Vilela N. Albuquerque P. Derengowski L. D. S. Silva-Pereira I. Kyaw C. M. (2013). Antibiotic development challenges: the various mechanisms of action of antimicrobial peptides and of bacterial resistance. Front. Microbiol. 4, 353. 10.3389/fmicb.2013.00353 24367355 PMC3856679

[B12] Jeszka-Skowron M. Krawczyk M. Zgoła-Grześkowiak A. (2015). Determination of antioxidant activity, rutin, quercetin, phenolic acids and trace elements in tea infusions: influence of citric acid addition on extraction of metals. J. Food Compos. Analysis 40, 70–77. 10.1016/j.jfca.2014.12.015

[B13] Kim D. H. Khan H. Ullah H. Hassan S. T. Šmejkal K. Efferth T. (2019). MicroRNA targeting by quercetin in cancer treatment and chemoprotection. Pharmacol. Res. 147, 104346. 10.1016/j.phrs.2019.104346 31295570

[B31] Kim G. S. Park C. R. Kim J. E. Kim H. K. Kim B. S. (2021). Anti-biofilm effects of torilis japonica ethanol extracts against *Staphylococcus aureus* . J. Microbiol. Biotechnol. 32 (2), 220–227. 10.4014/jmb.2107.07053 PMC9628846 34866130

[B14] Li Q. Cheng T. Wang Y. Bryant S. H. (2010). PubChem as a public resource for drug discovery. Drug Discov. today 15 (23-24), 1052–1057. 10.1016/j.drudis.2010.10.003 20970519 PMC3010383

[B32] Li S. Zhang W. Yang Y. Ma T. Guo J. Wang S. (2016). Discovery of oral-available resveratrol-caffeic acid based hybrids inhibiting acetylated and phosphorylated STAT3 protein. Europ. J. Medici. Chemis. 124, 1006–1018. 10.1016/j.ejmech.2016.10.028 27783972

[B16] Lipinski C. A. Lombardo F. Dominy B. W. Feeney P. J. (1997). Experimental and computational approaches to estimate solubility and permeability in drug discovery and development settings. Adv. drug Deliv. Rev. 23 (1-3), 3–25. 10.1016/S0169-409X(96)00423-1 11259830

[B17] Liu J. Du C. Beaman H. T. Monroe M. B. B. (2020). Characterization of phenolic acid antimicrobial and antioxidant structure–property relationships. Pharmaceutics 12 (5), 419. 10.3390/pharmaceutics12050419 32370227 PMC7285200

[B19] Muniesa M. Hammerl J. A. Hertwig S. Appel B. Brüssow H. (2012). Shiga toxin-producing *Escherichia coli* O104: H4: a new challenge for microbiology. Appl. Environ. Microbiol. 78 (12), 4065–4073. 10.1128/AEM.00217-12 22504816 PMC3370534

[B20] Najarzadeh Z. Mohammad-Beigi H. Nedergaard Pedersen J. Christiansen G. Sønderby T. V. Shojaosadati S. A. (2019). Plant polyphenols inhibit functional amyloid and biofilm formation in Pseudomonas strains by directing monomers to off-pathway oligomers. Biomolecules 9 (11), 659. 10.3390/biom9110659 31717821 PMC6920965

[B21] Qi D. Lu M. Li J. Ma C. (2023). Metabolomics reveals distinctive metabolic profiles and marker compounds of Camellia (Camellia sinensis L.) bee pollen. Foods 12 (14), 2661. 10.3390/foods12142661 37509753 PMC10378613

[B22] Shariati A. Noei M. Askarinia M. Khoshbayan A. Farahani A. Chegini Z. (2024). Inhibitory effect of natural compounds on *quorum* sensing system in *Pseudomonas aeruginosa*: a helpful promise for managing biofilm community. Front. Pharmacol. 15, 1350391. 10.3389/fphar.2024.1350391 38628638 PMC11019022

[B23] Tacconelli E. Carrara E. Savoldi A. Harbarth S. Mendelson M. Monnet D. L. (2018). Discovery, research, and development of new antibiotics: the WHO priority list of antibiotic-resistant bacteria and tuberculosis. Lancet Infect. Dis. 18 (3), 318–327. 10.1016/s1473-3099(17)30753-3 29276051

[B24] Tavares T. D. Antunes J. C. Padrão J. Ribeiro A. I. Zille A. Amorim M. T. P. (2020). Activity of specialized biomolecules against gram-positive and gram-negative bacteria. Antibiotics 9 (6), 314. 10.3390/antibiotics9060314 32526972 PMC7344598

[B26] Veiko A. G. Olchowik-Grabarek E. Sekowski S. Roszkowska A. Lapshina E. A. Dobrzynska I. (2023). Antimicrobial activity of quercetin, naringenin and catechin: flavonoids inhibit Staphylococcus aureus-induced hemolysis and modify membranes of bacteria and erythrocytes. Molecules 28 (3), 1252. 10.3390/molecules28031252 36770917 PMC9920354

[B27] Weese J. S. Prescott J. F. (2021). “Staphylococcal infections,” in Greene's infectious diseases of the dog and cat (WB Saunders), 611–626. 10.1016/B978-0-323-50934-3.00051-3

[B28] Zhao C. N. Tang G. Y. Cao S. Y. Xu X. Y. Gan R. Y. Liu Q. (2019). Phenolic profiles and antioxidant activities of 30 tea infusions from green, black, oolong, white, yellow and dark teas. Antioxidants 8 (7), 215. 10.3390/antiox8070215 31295859 PMC6680489

[B29] Zhao T. Li C. Wang S. Song X. (2022). Green tea (Camellia sinensis): a review of its phytochemistry, pharmacology, and toxicology. Molecules 27 (12), 3909. 10.3390/molecules27123909 35745040 PMC9231383

[B30] Zhao Z. Feng M. Wan J. Zheng X. Teng C. Xie X. (2021). Research progress of epigallocatechin-3-gallate (EGCG) on anti-pathogenic microbes and immune regulation activities. Food & Funct. 12 (20), 9607–9619. 10.1039/D1FO01352A 34549212

